# Association of EZSCAN Values with Arterial Stiffness in Individuals without Diabetes or Cardiovascular Disease

**DOI:** 10.1371/journal.pone.0090854

**Published:** 2014-03-03

**Authors:** Qiang Zeng, Sheng-Yong Dong, Man-Liu Wang, Hang Xiang, Xiao-Lan Zhao

**Affiliations:** 1 International Medical Center, Chinese PLA General Hospital, Beijing, China; 2 Center of Biomedical Analysis, Tsinghua University, Beijing, China; 3 Southwest Hospital, Third Military Medical University, Chongqing, China; INRCA, Italy

## Abstract

**Background:**

The EZSCAN test was recently developed to screen for early dysglycemia through an assessment of sudomotor function. Given the associations of dysglycemia and autonomic dysfunction with the development of arterial stiffness, EZSCAN may also detect early arterial stiffness. The aim of this study was to investigate the association of EZSCAN with arterial stiffness across blood glucose levels.

**Methodology and Principal Findings:**

A total of 5532 participants without diabetes or established cardiovascular disease were evaluated with EZSCAN. Their central systolic blood pressure (cSBP), brachial-ankle pulse wave velocity (baPWV), and ankle-brachial index (ABI) were also measured. Multivariate linear regression analyses were used to assess the association between the EZSCAN value and the cSBP, baPWV, and ABI measurements in all of the participants, with additional subgroup analysis that separated participants into a normal glucose tolerance (NGT) group and an impaired glucose regulation (IGR) group. The frequency of the IGRs increased with quartiles of the EZSCAN value (*P* for trend <0.0001). The levels of cSBP and baPWV increased while the levels of ABI decreased across quartiles of EZSCAN value in both NGT and IGR individuals (*P* for trend <0.0001 for all). In multivariable analyses, the EZSCAN value was positively associated with cSBP (log-transformed beta = 8.20, *P*<0.0001) and baPWV (log-transformed beta = 1.82, *P*<0.0001) but inversely associated with ABI (log-transformed beta = −0.043, *P*<0.0001) and was independent of conventional factors. Further adjustment for fasting and postprandial glucoses did not attenuate the associations. The results were also unchanged when stratified by IGR.

**Conclusions and Significance:**

The EZSCAN results were associated with arterial stiffness independent of conventional factors, blood glucose levels, and glucose tolerance status, suggesting a probable link between the EZSCAN value and arterial stiffness through autonomic dysfunction. The EZSCAN test may help us detect the development of arterial stiffness in high risk individuals to prevent unfavorable cardiovascular events.

## Introduction

Using reverse iontophoresis technology, the EZSCAN system is a noninvasive and accurate tool for the assessment of sudomotor function [1]. Because poor glucose control can injure autonomic nerve fibers and cause sudomotor dysfunction in patients with type 2 diabetes [2], the EZSCAN system has been tested in several countries as a tool for detecting diabetes and its complication of autonomic dysfunction [1], [3], [4]. Delivering rapid, simple, and reproducible measurements, the EZSCAN system has also been recently tested as a useful screening tool in high risk individuals for impaired glucose regulation (IGR) [5], [6].

Arterial stiffness is recognized as an important and independent risk factor for cardiovascular events. Identification of arterial stiffness has important implications for the primary prevention of future cardiac events in high risk individuals [7]. Due to the close association between increased arterial stiffness and dysglycemia [8], [9], the EZSCAN value may reflect arterial stiffness and therefore provide a new method for the detection of arterial stiffness. However, data are lacking on the relationship of EZSCAN results and arterial stiffness. Additionally, given the important link between arterial stiffness and autonomic dysfunction [10], [11], it is also unknown whether the association between the EZSCAN value and arterial stiffness is independent of blood glucose levels.

Therefore, in a cross-sectional study, we investigated the association of EZSCAN results with arterial stiffness in a large population without diabetes or established cardiovascular disease. We then stratified the individuals into normal glucose tolerance (NGT) and IGR groups to test whether differences in blood glucose levels would impact this association.

## Methods

### Ethics Statement

This study was approved by the Medical Ethic Committee of Chinese People's Liberation Army General Hospital. Informed written consent was obtained from all participants.

### Study population

The individuals who were recruited into the study population had presented to Chinese PLA General Hospital (Beijing, China) and had undergone a routine physical examination between April 2012 and January 2013. Subjects were included if they were over 18 years old. Individuals with previously known cardiovascular disease, diabetes, chronic kidney disease with an estimated glomerular filtration rate (GFR) <60 ml/min, cancer, severe psychiatric disturbance, or were pregnant were excluded. A total of 5680 individuals were invited to complete standardized questionnaires, an EZSCAN test, and an assessment of arterial stiffness. After further excluding 31 individuals with incomplete demographic information or physical examinations, 94 individuals with fasting glucose ≥7.0 mmol/l and/or postprandial glucose ≥11.1 mmol/l; and 23 individuals with using antihypertensive, cholesterol-lowering, or glucose-lowering medications, 5532 participants were ultimately included in the study.

### Data collection

Demographic characteristics, smoking status, and individual and family medical history were obtained by a standard questionnaire. Weight was measured to the nearest 0.1 kg and height to the nearest 0.1 cm. Body mass index was calculated as weight in kilograms divided by the square of height in meters. Waist circumference was measured at the midpoint between the lower ribs and the pelvic bone. The heart rate was taken in a sitting position after 10 minutes of rest. Resting systolic and diastolic blood pressures were measured using a manual sphygmomanometer. The heart rate and the blood pressure were measured twice and the means of the 2 measurements were used for analysis. All participants took a 75-g oral glucose tolerance test, and blood samples were collected at 0 and 2 hours to measure the fasting and postprandial plasma glucose levels. Triglyceride, high-density lipoprotein cholesterol (HDL cholesterol), blood glucose, and creatinine were measured as previously described [12], [13], [14]. The estimated GFR was calculated using the Modification of Diet in Renal Disease equation [15]. All tests were performed by trained personnel blinded to the data.

In accordance with the 1999 World Health Organization diagnostic criteria [16], NGT was defined as a fasting plasma glucose <6.1 mmol/l and postprandial plasma glucose <7.8 mmol/l, while IGR was defined as either an impaired fasting glucose (fasting plasma glucose ≥6.1 mmol/l) and/or an impaired glucose tolerance (postprandial plasma glucose ≥7.8 mmol/l).

### EZSCAN test

The EZSCAN device (Impeto Medical, Paris, France) is designed to accurately evaluate sweat gland function with reverse iontophoresis and chronoamperometry. The measurement was detailedly described in previously published studies [3], [6]. Briefly, EZSCAN measures electrochemical skin conductance that is a result of the reaction between the chloride ions in the sweat and the nickel electrodes of the device. During the test, six nickel electrodes were placed on the hands, feet, and forehead, where the skin is rich in sweat glands. A direct current at an incremental voltage of ≤4 V was applied to the electrodes. The electrochemical skin conductance was calculated using the ratio of the current measured over the constant power applied. The EZSCAN value was then calculated with an algorithm that accounts for different parameters, including the electrochemical skin conductance of hands, feet, and forehead and demographic data, such as sex, age, height, weight and systolic blood pressure. The EZSCAN value ranges from 0 to 100%. A higher EZSCAN value indicates a higher risk of sudomotor dysfunction.

### Assessment of arterial stiffness

Markers of arterial stiffness that included the central systolic blood pressure (cSBP), the brachial-ankle pulse wave velocity (baPWV), and the ankle-brachial index (ABI) were measured in the supine position after a 10 minutes rest. Applanation tonometry of the radial artery with a high-fidelity transducer (Millar Instruments, Houston, Texas) was used to obtain an averaged radial pulse. The radial artery pressure waveform was calibrated to a supine brachial blood pressure. The inbuilt transfer function in the SphygmoCor system (Atcor, West Ryde, Australia) provided a corresponding aortic pulse waveform from which cSBP was identified [17]. The baPWV was calculated using a volume plethysmograph (PP1100, Hanbyul Meditech, Hong Kong), as described previously [14]. Both ankle and brachial systolic blood pressure were measured, and the ABI was calculated as the ankle systolic blood pressure divided by the brachial systolic blood pressure. Individuals with ABI values higher than 1.3 were considered to have a non-compressible vessel and were excluded from the analysis. All recordings were performed on the right side of the body. All of the measurements were performed in triplicate by trained investigators, and the mean values were used for analysis.

### Statistical analysis

All normally distributed variables are expressed as the mean ± standard deviation (SD). Data that were not normally distributed are expressed as the median (interquartile range). Categorical variables are expressed as percentages. The Kolmogorov-Smirnov test was used to evaluate data for normal distribution. Baseline demographics were stratified by IGR status and the EZSCAN value. Normal variables were compared using the Student *t* test, and non-normal variables were logistically transformed and compared using the Student *t* test. Categorical variables were compared using the chi-square test. The frequency of IGR across quartiles of EZSCAN values was compared using the chi-square test, and the *P* for trend was calculated. Spearman's correlation coefficients between the EZSCAN value and arterial stiffness indices were calculated in NGT and IGR individuals separately. To further examine the association between the EZSCAN value and arterial stiffness in NGT and IGR individuals, one-way analysis of variance and the *P* for trend were first used to compare the levels of cSBP, baPWV, and ABI across the quartiles of the EZSCAN value; unadjusted and adjusted multivariate logistic regression analyses were then performed. Model 1 is unadjusted. Model 2 is adjusted for age, sex, smoking, heart rate, body mass index, and waist circumference. Model 3 is adjusted for all covariates from Model 2 plus systolic blood pressure, diastolic blood pressure, triglycerides, HDL cholesterol, and estimated GFR. Model 4 is further adjusted for fasting glucose and postprandial glucose. EZSCAN values were logistically transformed in all models. In model 3 and model 4, the variable of systolic blood pressure was not included when the dependent variable was cSBP; the variables of systolic and diastolic blood pressures were replaced by the variable of mean arterial pressure when the dependent variable was baPWV. All analyses were performed using SPSS version 10.0 (SPSS Inc., Chicago, Illinois), and a two-sided *P* value <0.05 was considered statistically significant.

## Results

### Baseline characteristics by IGR status and EZSCAN value

**Figure 1:**
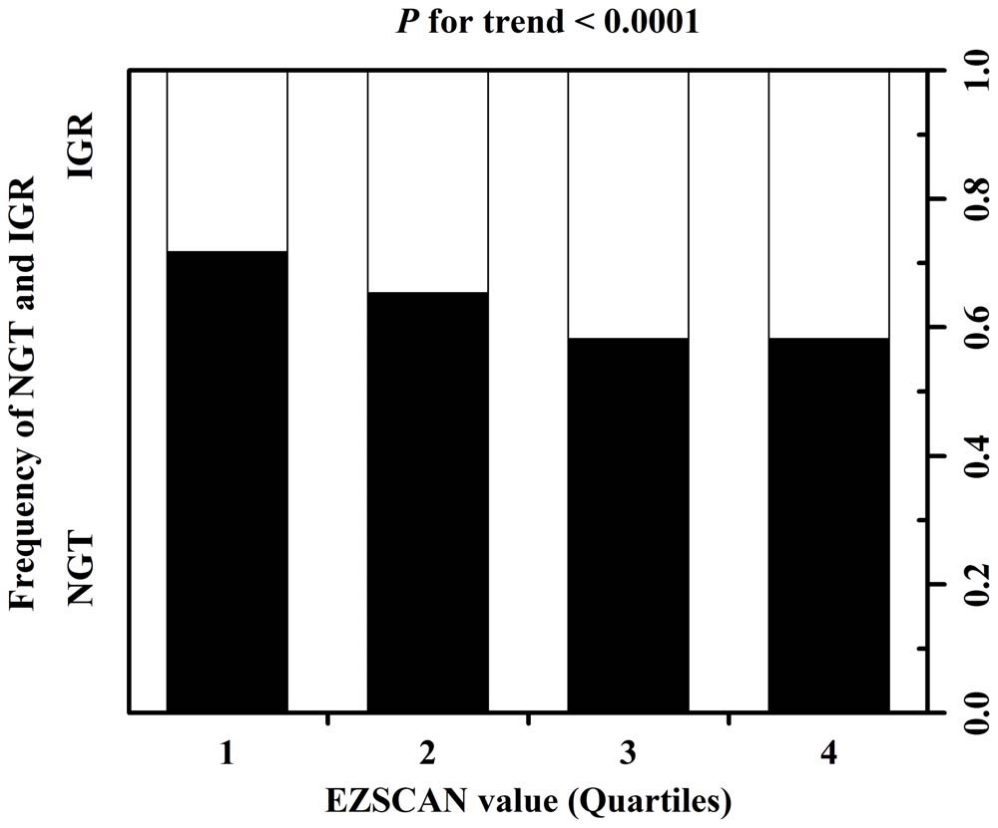
Prevalence of NGT (solid bars) and IGR (open bars) across EZSCAN value quartiles.

Baseline characteristics of the study population are shown in **Table 1**. Among the 5532 participants meeting study criteria, the mean age was 48.0, 34.4% was female, 37.0% had IGR, and 52.8% had an EZSCAN value higher than the median EZSCAN value of 26. Compared with individuals without IGR, individuals with IGR were older, more often male, with a greater body mass index, waist circumference, systolic and diastolic blood pressures, triglycerides, HDL cholesterol, fasting and postprandial glucoses, and EZSCAN value (*P*<0.05 for all). Similar results were found when comparing individuals with an EZSCAN value above the median with those with a value below the median; however, there was no difference in the gender distribution between the two groups, and the estimated GFR was lower in individuals with an EZSCAN value above the median than in those with a value below the median (*P*<0.05). In addition, there was a statistically significant increase in prevalence of IGR across the quartiles of the EZSCAN value (28.3%, 34.7%, 41.8%, and 41.8% for the 1^st^, 2^nd^, 3^rd^, and 4^th^ quartiles, respectively, *P* for trend <0.0001) (**Figure 1**).

**Table 1:** Baseline characteristics, stratified by IGR status and the EZSCAN value.

	Glucose regulation status		EZSCAN value	
Variable	NGT	IGR	< Median	≥ Median
	(n = 3486)	(n = 2046)	(n = 2611)	(n = 2921)
Age, years	46.5±8.8	50.6±8.7*	44.6±8.1	51.0±8.7†
Male	2244 (64.4)	1384 (67.6)*	1698 (65.0)	1930 (66.1)
Body mass index, kg/m^2^	25.0±3.4	25.9±3.3*	24.5±3.2	26.0±3.4†
Waist circumference, cm	85.5±10.5	88.5±10.0*	84.2±10.2	88.7±10.1†
Current smoking	1117 (32.0)	628 (30.7)	799 (30.6)	946 (32.4)
Family history of diabetes	741 (21.3)	435 (21.3)	571 (21.9)	605 (20.7)
Family history of CVD	737 (21.1)	406 (19.8)	541 (20.7)	602 (20.6)
Heart rate	70±10	71±10*	70±10	71±10†
Systolic blood pressure, mmHg	119±17	126±19*	114±14	128±18†
Diastolic blood pressure, mmHg	79±12	82±12*	77±10	83±12†
Mean arterial pressure, mmHg	92±13	97±14*	89±11	98±14†
Triglycerides, mmol/l	1.78±1.67	2.15±2.01*	1.84±1.78	1.98±1.84†
HDL cholesterol, mmol/l	1.25±0.34	1.19±0.32*	1.24±0.34	1.21±0.33†
Fasting glucose, mmol/l	5.24±0.41	5.99±1.30*	5.40±0.80	5.62±1.02†
Postprandial glucose, mmol/l	6.19±0.93	9.17±2.12*	7.02±1.87	7.54±2.20†
Estimated GFR, ml/min per 1.73 m^2^	111 (98–127)	112 (99–127)	114 (101–129)	110 (97–126)†
EZSCAN value, %	25 (23–45)	27 (24–50)*	24 (22–24)	45 (28–54)†

Values are mean ± SD, n (%), or median (interquartile range). Normal variables were compared using the Student t test, and non-normal variables were logistically transformed and compared using the Student t test. Categorical variables were compared using the chi-square test.

NGT, normal glucose tolerance; IGR, impaired glucose regulation; CVD, cardiovascular disease; HDL cholesterol, high-density lipoprotein cholesterol; GFR, glomerular filtration rate.

^*^ *P*<0.05 vs. NGT group.

† *P*<0.05 vs. < Median EZSCAN value group.

### Association of EZSCAN value with arterial stiffness

Spearman's correlation coefficients between the EZSCAN value and arterial stiffness indices are presented in **Table 2**. The EZSCAN value was positively related to the cSBP (Spearman's correlation coefficients  = 0.253) and the baPWV (Spearman's correlation coefficients  = 0.243); but was negatively related to the ABI (Spearman's correlation coefficients  = −0.167) (*P*<0.0001 for all). These relationships between the EZSCAN value and markers of arterial stiffness remained significant even when the individuals were then stratified by IGR status (*P*<0.0001 for all).

**Table 2:** Spearman's correlation coefficients between the EZSCAN value and indices of arterial stiffness.

	EZSCAN value	Individuals with NGT	Individuals with IGR
		EZSCAN value	EZSCAN value
cSBP	0.253***	0.235***	0.249***
baPWV	0.243***	0.231***	0.230***
ABI	−0.167***	−0.181***	−0.123***

cSBP, central systolic blood pressure; baPWV, brachial-ankle pulse wave velocity; ABI, ankle-brachial index.

^***^ *P*<0.0001; ^**^ *P*<0.01; ^*^ *P*<0.05.

**Figure 2:**
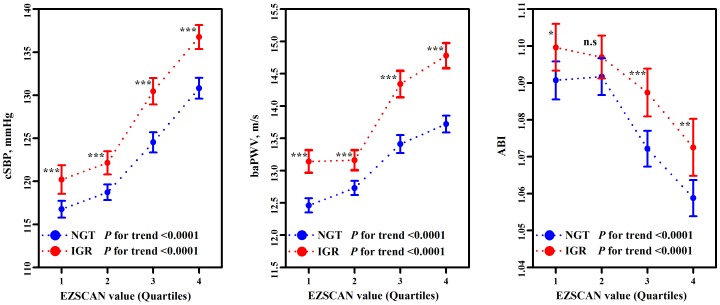
Arterial stiffness across the EZSCAN value quartiles, stratified by IGR status. Error bars represent 95% CI of the mean. n.s, not significant. ^***^P<0.0001; ^**^ P<0.01; ^*^ P<0.05.

To further investigate the association between the EZSCAN value and arterial stiffness, the comparison of the cSBP, baPWV, and ABI were performed across the quartiles of the EZSCAN value. The means of both cSBP and baPWV increased across the EZSCAN value quartiles, while the means of ABI decreased with each EZSCAN value quartile (*P* for trend <0.0001 for all) (**Table 3**). When individuals were stratified by IGR status, the means of the cSBP and baPWV continued to increase, while the means of ABI continued to decrease across the quartiles of the EZSCAN value in both NGT and IGR individuals (*P* for trend <0.0001 for all) (**Figure 2**). Furthermore, the means of cSBP, baPWV, and ABI were higher in individuals with IGR than in individuals with NGT across all quartiles of the EZSCAN value (*P*<0.05 for all), except for the ABI mean in the 2^nd^ quartile of the EZSCAN value.

**Table 3:** Means of arterial stiffness indices across the EZSCAN value quartiles.

	The first quartile	The second quartile	The third quartile	The fourth quartile	*P* for trend
cSBP, mmHg	118 (117–119)	120 (119–121)	127 (126–128)	133 (132–134)	<0.0001
baPWV, m/s	12.7 (12.6–12.7)	12.9 (12.8–13.0)	13.8 (13.7–13.9)	14.2 (14.0–14.3)	<0.0001
ABI	1.09 (1.09–1.10)	1.09 (1.09–1.10)	1.08 (1.07–1.08)	1.06 (1.06–1.07)	<0.0001

Values are mean (95% CI).

Multivariate regression analyses of the association between the EZSCAN value and arterial stiffness indices are shown in **Table 4**. In the unadjusted regression models, the EZSCAN value was positively associated with cSBP (log-transformed beta = 17.08, *P*<0.0001) and baPWV (log-transformed beta = 2.29, *P*<0.0001). After adjustment for age, sex, smoking, heart rate, body mass index, and waist circumference, the EZSCAN value remained positively associated with cSBP (log-transformed beta = 10.91, *P*<0.0001) and baPWV(log-transformed beta = 2.27, *P*<0.0001). After further adjustment for diastolic blood pressure (in the cSBP model) or mean arterial pressure (in the baPWV model), triglycerides, HDL cholesterol, and estimated GFR, the positive associations of the EZSCAN value with cSBP (log-transformed beta = 8.20, *P*<0.0001) and baPWV (log-transformed beta = 1.82, *P*<0.0001) were unchanged. Furthermore, additional adjustment for fasting glucose and postprandial glucose did not attenuate the associations. In contrast, the EZSCAN value was inversely associated with ABI in all regression models (log-transformed betas were −0.061, −0.051, −0.043, and −0.027 in the unadjusted model 1, tmodel 2, model 3, and model 4, respectively; *P*<0.0001 for all). The associations of the EZSCAN value with the cSBP, baPWV, and ABI remained significant when individuals were stratified by IGR status in all regression models (*P*<0.05 for all).

**Table 4:** Multivariate linear regression models of the relationship between the EZSCAN value and arterial stiffness indices.

	cSBP		baPWV		ABI	
	Beta	*P* value	Beta	*P* value	Beta	*P* value
All individuals						
Model 1 (unadjusted)	17.08	<0.0001	2.29	<0.0001	−0.061	<0.0001
Model 2	10.91	<0.0001	2.27	<0.0001	−0.051	<0.0001
Model 3*	8.20	<0.0001	1.82	<0.0001	−0.043	<0.0001
Model 4*	7.00	<0.0001	1.62	<0.0001	−0.027	<0.0001
Individuals with NGT						
Model 1 (unadjusted)	15.16	<0.0001	1.92	<0.0001	−0.066	<0.0001
Model 2	9.41	<0.0001	1.97	<0.0001	−0.053	<0.0001
Model 3*	7.68	<0.0001	1.68	<0.0001	−0.048	<0.0001
Model 4*	7.15	<0.0001	1.60	<0.0001	−0.028	0.0004
Individuals with IGR						
Model 1 (unadjusted)	16.90	<0.0001	2.39	<0.0001	−0.042	<0.0001
Model 2	11.52	<0.0001	2.41	<0.0001	−0.038	0.0001
Model 3*	7.60	<0.0001	1.74	<0.0001	−0.030	0.003
Model 4*	6.48	0.0007	1.59	<0.0001	−0.026	0.021

Model 1 is unadjusted. Model 2 is adjusted for age, sex, smoking, heart rate, body mass index, and waist circumference. Model 3 is adjusted for all covariates from Model 2 plus systolic blood pressure, diastolic blood pressure, triglycerides, HDL cholesterol, and estimated GFR. Model 4 is further adjusted for fasting glucose and postprandial glucose.

*In these models, the variable of systolic blood pressure was not included when the dependent variable was cSBP; the variables of systolic and diastolic blood pressures were replaced by the variable of mean arterial pressure when the dependent variable was baPWV.

## Discussion

In this large, population-based, cross-sectional study, the EZSCAN value was associated with arterial stiffness indices, independent of conventional factors and blood glucose. Accounting for glucose tolerance status did not attenuate this association, suggesting that the link between the EZSCAN value and arterial stiffness may be at least partially independent of glucose levels and glucose regulation in individuals without established diabetes or cardiovascular disease. These results highlight the potential of the EZSCAN test as a new tool for the detection of arterial stiffness in high risk individuals and facilitate the primary prevention of cardiovascular events.

As a new method for assessing sudomotor and autonomic function [1], [3], the EZSCAN test reportedly has a good sensitivity and specificity for screening diabetes and impaired glucose regulation [4], [5], [6]. Recently, the EZSCAN test has also been reported as a simple method for early assessment of the metabolic syndrome [18]. The link between the EZSCAN value and diabetes or the metabolic syndrome has been explained as an autonomic dysfunction resulting from dysglycemia or metabolic dysfunction [6], [18], [19]. In the present study, we observed an association between the EZSCAN value and arterial stiffness indices. Considering the closing relationships of dysglycemia and metabolic dysfunction with arterial stiffness [9], [20], our study further stratified individuals into NGT and IGR groups and analyzed the findings in several regression models. The findings in the models were independent of blood glucose levels and conventional factors and were consistent in NGT and IGR subgroups. Our observations provide insight into the potential relationship between the EZSCAN value and arterial stiffness, independent of blood glucose levels, supporting the hypothesis that autonomic dysfunction, as reflected by the EZSCAN value, may be associated with the development of arterial stiffness.

Few studies have examined the relationship between autonomic dysfunction and arterial stiffness. We speculate that this relationship is bidirectional. A study of 25 healthy male subjects demonstrated a positive association between muscle sympathetic nerve activity and PWV [10]. Sympathetic overactivity in the short term can reduce arterial compliance through changes in arterial smooth muscle tone and may also play an important mechanistic role in arterial stiffness in the longer term [21]. Moreover, increased sympathetic activity promotes vascular muscle growth and arterial wall fibrosis either directly or by its effects on the renin-angiotensin-aldosterone system, and consequently contributes to arterial stiffness [22], [23]. Meanwhile, the development of arterial stiffness may in return mediate, at least in part, chronic sympathetic nerve activity. Large artery stiffness can interfere with autonomic regulation by impairing the sensitivity of the carotid baroreflex [24]. In individuals with high risk of arterial stiffness, endothelial dysfunction and several other factors regulating vascular function, such as nitric oxide, reactive oxygen species, and endothelin also interact with the sympathetic nervous system [25], [26].

Increased arterial stiffness is associated with left ventricular hypertrophy, reduced coronary perfusion with aggravation of ischemia and progressive atherosclerosis, and increased risk of cardiovascular events [20], [27], [28]. Early detection of arterial stiffness may identify individuals with a high cardiovascular risk and help facilitate the primary prevention of cardiovascular events. Our analysis was limited to individuals without diabetes or cardiovascular disease to focus on the early detection of arterial stiffness. In addition, the cSBP, PWV, and ABI have been proved as independent predictors of cardiovascular events [29], [30]. These markers can now be measured easily and non-invasively, most likely becoming the most widely used method for the assessment of arterial stiffness [20], [31]. Considering that autonomic function and vascular function are both key factors in the development and prognosis of cardiovascular events and disease [32], [33], and that increased sympathetic activation in individuals with arterial stiffness worsens the prognosis in this high-risk population [21], we used a test of autonomic function to assess arterial stiffness and analyzed the associations of the EZSCAN value with markers of arterial stiffness. Our findings suggest that the EZSCAN test, as a simple tool, can provide additional information about autonomic dysfunction for the early detection and management of arterial stiffness.

In the study, the PWV was measured using baPWV rather than carotid-femoral PWV. The carotid-femoral PWV is considered the gold-standard method for assessing aortic stiffness and predicts future cardiovascular events and all-cause mortality [29], [34], [35], but baPWV has been increasingly used in the research and clinical settings because its measurement is extremely simple, allowing repeated measurements in a large number of study subjects [36]. Using 18 longitudinal cohort studies, Vlachopoulos et al. has reported that baPWV is associated with increased risk of total cardiovascular events and all-cause mortality [37]. Furthermore, the value of PWV is closely related to age [34]. The mean of baPWV in our study (12.7 to 14.2 m/s) was slightly higher than a prospective study in Japan (12.3 to 14.0 m/s) [36], but lower than another general middle and aged population-based study in China (the median of baPWV was 15.1 m/s) [38]. This variation can be partly explained by the difference of the mean age between subjects in our study (48 years) and subjects in the Japanese study (40 years) and the other Chinese study (58 years) [36], [38].

Interestingly, in the present study, we also observed that subjects with an EZSCAN value above the median had a lower estimated GFR than their counterparts with an EZSCAN value below the median. Considering that elevated sympathetic activity is much more frequent in chronic kidney disease [39], further studies are needed to evaluate the possibility of the EZSCAN test as a tool to provide information about autonomic dysfunction of the early renal dysfunction.

Strengths of the current study include a large-scale, population-based design and a novel technology-based assessment of arterial stiffness using the EZSCAN system. Several limitations also merit comment. Because the analysis was cross-sectional, we are unable to determine the causal relationship between autonomic function and arterial stiffness beyond what has been suggested from experimental data; therefore, the findings of this study require further confirmation in prospective studies. Secondly, although the EZSCAN test has been validated as a reliable tool for the assessment of sudomotor and autonomic function, other available techniques for assessing sudomotor and autonomic function, such as the quantitative sudomotor axon reflex test [6] and microneurography [10], should be used to reassess our findings.

In conclusion, we demonstrated an association between the EZSCAN value and arterial stiffness that is independent of conventional risk factors and blood glucose levels in individuals without diabetes or cardiovascular disease. The findings were consistent in the NGT and IGR subgroup analysis. These results support the hypothesis that autonomic dysfunction, reflected by the EZSCAN value, may be associated with the development of arterial stiffness, suggesting that the EZSCAN test can be used as a new tool for the detection and primary prevention of early arterial stiffness in high risk individuals.
